# Physiological, Biochemical, Epigenetic and Molecular Analyses of Wheat (*Triticum aestivum*) Genotypes with Contrasting Salt Tolerance

**DOI:** 10.3389/fpls.2017.01151

**Published:** 2017-06-30

**Authors:** Suresh Kumar, A. S. Beena, Monika Awana, Archana Singh

**Affiliations:** Division of Biochemistry, ICAR – Indian Agricultural Research InstituteNew Delhi, India

**Keywords:** antioxidant potential, epigenetics, high-affinity potassium transporter, membrane stability, salt tolerance, sodium chloride

## Abstract

Abiotic stress exerts significant impact on plant’s growth, development, and productivity. Productivity of crop plants under salt stress is lagging behind because of our limited knowledge about physiological, biochemical, epigenetic, and molecular mechanisms of salt tolerance in plants. This study aimed to investigate physio-biochemical, molecular indices and defense responses of selected wheat cultivars to identify the most contrasting salt-responsive genotypes and the mechanisms associated with their differential responses. Physio-biochemical traits specifically membrane stability index, antioxidant potential, osmoprotectants and chlorophyll contents, measured at vegetative stage, were used for multivariate analysis to identify the most contrasting genotypes. Genetic and epigenetic analyses indicated the possible mechanisms associated with differential response of the wheat genotypes under salt stress. Better antioxidant potential, membrane stability, increased accumulation of osmolytes/phytophenolics, and higher K^+^/Na^+^ ratio under 200 mM NaCl stress identified Kharchia-65 to be the most salt-tolerant cultivar. By contrast, increased MDA level, reduced soluble sugar, proline, total chlorophyll, total phenolics contents, and lower antioxidant potential in HD-2329 marked it to be sensitive to the stress. Genetic and bioinformatics analyses of *HKT1;4* of contrasting genotypes (Kharchia-65 and HD-2329) revealed deletions, transitions, and transversions resulting into altered structure, loss of conserved motifs (Ser-Gly-Gly-Gly and Gly-Arg) and function in salt-sensitive (HD-2329) genotype. Expression analysis of HKTs rationalized the observed responses. Epigenetic variations in cytosine methylation explained tissue- and genotype-specific differential expression of *HKT2;1* and *HKT2;3*.

## Introduction

Wheat is one of the most important cereal crops having global production of >700 million tones, and provides 20% of the daily protein requirements, and calories for 4.5 billion people globally (Arzani and Ashraf, 2017). Limited success in growing wheat on salt-affected soils has been achieved because only a few salt-tolerant bread wheat genotypes have been identified (Sairam et al., 2002). Kharchia-65 (a collection from Kharchi in Pali district of Rajasthan, India) is one of the very few bread wheat genotypes showing tolerance to salinity and sodicity stresses. KRL-210 is another released variety (developed at Central Soil Salinity Research Institute, Karnal, India) and reported to be most salt-tolerant variety among the KRL series (Kumar et al., 2015). Though, KRL-210 has been reported to be agronomically superior (semi-dwarf, higher grain yield) over Kharchia-65 under normal conditions, their comparative salt tolerance ability has not been reported so far. Salt stress not only reduce the expected yield of crops but also affects metabolic processes in plants through impairment of water potential of cells, ion toxicity, membrane integrity and function, and uptake of essential mineral nutrients (Arzani and Ashraf, 2016). Investigations on stress responses of plants have been the focus of the breeders for a long time, but insight into the stress perception and signaling has recently been complemented by the growing evidences for stress-induced biochemical, physiological and epigenetic changes (Kumar and Singh, 2016; Kumar et al., 2017). An enhanced knowledge of about biochemical, physiological, genetic and epigenetic aspects of salt tolerance will not only be helpful in cloning of the genes involved in salt tolerance, development of transgenics and better breeding programs, but also in screening germplasm toward breeding for saline conditions (Sairam et al., 2002).

One of the detrimental effects of salinity is the accumulation of sodium ion (Na^+^) in plant tissues. Higher concentration of Na^+^ inhibits uptake of essential macronutrients like potassium (K^+^) and calcium (Ca^2+^) from soil (Very and Sentenac, 2003). K^+^ is an essential macronutrient for growth/development of plant and for maintaining high K^+^/Na^+^ ratio in shoot, which has been suggested to be a major strategy adopted by plants to cope up with salt stress (Hamamoto et al., 2015). Though Na^+^ and K^+^ have similar chemical properties and content ratio in non-saline soils, physiological impacts of these ions on metabolism and growth of plants are quite different. At cellular level, different mechanisms for salt tolerance function to reduce Na^+^ accumulation in the cytoplasm by limiting Na^+^ entry into the cell, actively transporting Na^+^ out of the cell, and compartmentalizing Na^+^ into the vacuole (Shi et al., 2003). Generally, K^+^ is preferred for uptake into roots from the soil and most plants show a high degree of K^+^/Na^+^ discrimination for their uptake. High-affinity potassium transporters (HKTs) are active at plasma membrane level and function as Na^+^/K^+^ symporter as well as Na^+^ selective uniporter (Horie et al., 2009). Phylogenetic analysis of HKTs revealed two major subfamilies, namely HKT1 and HKT2. It was proposed by Platten et al. (2006) that the transporters of HKT1;x subfamily are permeable to Na^+^ only, whereas transporters of the subfamily HKT2;y are permeable to both Na^+^ and K^+^. The families of HKTs belong to HKT/Trk/Ktr-type K^+^ transporter superfamily which are found in microorganisms and plants (Yamaguchi et al., 2013). Downregulated expression of *TaHKT2* genes in salt-tolerant wheat genotype was reported to impart tolerance to salt (NaCl) stress (Singh et al., 2015). However, understanding the molecular mechanism(s) of gene regulation is equally important for engineering wheat for increased tolerance (Arzani and Ashraf, 2016). Although Na^+^ exclusion leading to better salinity tolerance is challenged by some of the researchers, *HKTs* have emerged as important component of salt stress tolerance in plants. The paradox may be explained by simultaneous differences and natural variation in other components that underpin salt tolerance, such as the ability to tolerate the effects of salt accumulation in shoot or to cope with the osmotic components of salinity (Waters et al., 2013).

Screening of wheat germplasm for salt tolerance has been carried out by many researchers and significant variation in their tolerance level has been reported. Field screening might be misleading due to complicated genotype × environment interaction and variability of the salt-affected filed (Arzani, 2008). Therefore, evaluation of plant’s ability to cope with salt stress may be more accurate if a combination of agronomical, biochemical, and physiological parameters is considered and the experiment is carried out under controlled conditions. Reactive oxygen species (ROS), produced during the stress cause chlorophyll degradation and membrane-lipid peroxidation. Malondialdehyde (MDA) is one of the final products of peroxidation of polyunsaturated fatty acids in the cell membranes. An increase in free-radicals causes overproduction of MDA which is commonly known as a marker of the oxidative stress. On exposure to stressful conditions, plants accumulate an array of metabolites. Soluble sugar, proline, phenolic compounds, chlorophyll contents, K^+^/Na^+^, shoot-root biomass ratios, etc. show significant changes under abiotic stress and they are considered important indicators of salt tolerance ability of plant. Total soluble sugar (TSS) content is not only a main photosynthate in plant, but also a main component of carbohydrate metabolism. Thus, soluble sugar content shows a close relationship between photosynthesis and plant’s productivity, and reflects the ability of leaves (and later on capability of the grains) to use assimilates. Proline, the only amino acid (actually imino acid containing a secondary amine group), not only acts as excellent osmolyte but also serves as metal chelator, antioxidative defense molecule and signaling molecule. Thereby, it maintains osmotic balance, membrane integrity and concentrations of ROS within a normal range; thus preventing oxidative burst in plant. Phenolic compounds, in plants and their produce, play important role in antioxidant activities to neutralize free-radicals, quenching singlet oxygen, and decomposing peroxides (Valifard et al., 2014). Synthesis of polyphenolic compounds and their accumulation are generally stimulated in response to abiotic/biotic stresses.

Salt stress causes metabolic damage, leaf senescence followed by photosynthetic decline leading to reduced plant productivity. These parameters have been successfully utilized in screening and/or evaluating salt tolerance ability of plants (Kumar et al., 2015; Singh et al., 2015). Objectives of the present study were to investigate physio-biochemical responses of four wheat cultivars under salt stress, and comprehensive evaluation of the genotypes for their salt tolerance level to identify the most contrasting salt-responsive genotypes. Mechanisms responsible for genotype- and tissue-specific differential expression of *TaHKTs* genes were also investigated.

## Materials and Methods

Mature and healthy seeds of Kharchia-65, KRL-210, HD-2329, and WH-542 were procured from ICAR-Indian Institute of Wheat and Barley Research, Karnal, India. The seeds were surface sterilized following the protocol described by Singh et al. (2015). Six seedlings were grown in a 15 cm pot filled with agro-coir peat at equal distance in triplicate. Seven pots for each genotype were grown under controlled conditions (25°C, 60 ± 10% RH, 16 h photoperiod) in a glasshouse at National Phytotron Facility, IARI, New Delhi. Two-week-old seedlings were subjected to salt stress by irrigating them with 0.5x Hoagland solution containing varying concentration (0, 50, 100, 150, 200, 250, and 300 mM) of NaCl. Shoot and root samples were collected at 0, 3, 6, 9, 12, 14, and 16 days after treatment (DAT) for biochemical and physiological analyses.

Based on the results of preliminary experiments (Supplementary Table S1) stress treatment with 200 mM NaCl for 14 days was used in the subsequent experiments. To study the effects of the salt stress on growth and development (morphology) of wheat genotypes, plant height, number of tillers and leaf senescence were recorded (**Table 1**). Estimation of leaf-area was carried out by randomly selecting plants from the replicated experiment using a leaf-area meter. Fresh weight (FW) of shoot and root of the randomly selected plant was determined immediately, and then samples were dried in oven at 80°C until no reduction in biomass was recorded to obtain dry weight (DW). Total chlorophyll content in leaf tissues (0.5 g) was estimated using dimethyl sulfoxide (DMSO) method as described by Hiscox and Israelstam (1979). Absorbance of the extract was taken at 645 and 663 nm against DMSO blank using spectrophotometer. Total chlorophyll content was calculated on the basis of FW using the following formula:

**Table 1:** Comparison of wheat cultivars for plant height, number of tillers, leaf senescence affected by salt stress (200 mM NaCl for 14 days).

Genotype	Plant height (cm)			Tillers per plant (No.)			Leaf senescence^∗^ (No.)	
	Control	Salt stress	% change^Ψ^	Control	Salt stress	% change^Ψ^	Control	Salt stress
KRL-210	79.59	60.74	23.68^c^	3.66	1.99	45.62^b^	0.00	2.33^b^
Kharchia-65	91.68	77.21	15.78^a^	3.33	2.33	30.03^a^	0.33^a^	1.66^a^
HD-2329	71.16	53.27	25.14^c^	2.66	1.33	50.00^c^	0.66^b^	5.33^c^
WH-542	70.32	56.43	19.75^b^	2.99	1.66	44.48^b^	0.00	2.66^b^

*^Ψ^Means followed by different *lowercase letters* in a *column* are significantly different (*P* ≤ 0.05) by Fisher’s least significant difference (LSD) test. ^∗^The number of lower leaves showing ≥50% senescence/drying. The values are means of three replications, and the experiment was repeated three times.*

$$\text{Total}\;\text{chlorophyll}\;(\text{mg/gFW})\;\text{= }(\text{20}.\text{2}\;\times \;{\text{A}}_{\text{645}})\;\text{+}\;(\text{8}.\text{02}\;\times \;{\text{A}}_{\text{663}}).$$

For estimation of lipid peroxidation (in terms of MDA level, a product of lipid peroxidation), sample extracts were prepared using 1.0 g fresh tissue following the procedure described by Cakmak and Horst (1991). Absorbance of the extract was recorded at 532 and 600 nm. Mean of the readings in triplicate was used to calculate MDA level with extinction coefficient of 155 mM^-1^ cm^-1^ and the formula:

$$\text{MDA}\;(\text{nM})\;\text{=}\;\Delta {\text{A}}_{(\text{532}}{{}_{\text{-}}}_{\text{600})}\text{/1}.\text{56}\;\times \;{\text{10}}^{\text{5}}.$$

Electrolyte leakage was estimated as described by Bhushan et al. (2007). Tissue samples were prepared and submersed in sterile distilled water in two sets with three replications. The first set of the samples was incubated at room temperature for 4 h, and then electrical conductivity (EC_1_) was recorded. The second set was autoclaved for 15 min (to release all ions from the tissues), cooled to 25°C and then the conductivity (EC_2_) was measured. The membrane stability index (MSI) was calculated using the formula:

$$\text{MSI}\;\text{=}\;[\text{1}\;\text{-}\;({\text{EC}}_{\text{1}}{\text{/EC}}_{\text{2}})]\;\times \;\text{100}.$$

Total soluble sugar was assayed by anthrone method which is widely used for determination of starch and soluble sugars. Absorbance of the extract was taken at 630 nm within 1 h using spectrophotometer. Standard curve of glucose/starch (0–10 mg/10 mL) was prepared by plotting concentration of glucose/starch on *X*-axis and spectrophotometer (A_630_) reading on *Y*-axis. Using the standard curve, concentration of glucose/starch in the sample tissues was calculated and expressed as μM/g DW. Proline content was determined according to the method of Bates et al. (1973) using Ninhydrin reagent. Proline-ninhydrin chromophore was extracted with 4.0 mL of toluene and absorbance was recorded at 520 nm. Proline content was calculated using the formula:

Proline (μM/g DW) = [(μg Proline/mL × mL Toluene)/ 115.5 μg/μM]/[(g sample)/5].

To estimate variation in production of phytophenolics induced by salt stress, total phenolics content (TPC) was estimated following the procedure of Singleton et al. (1999). Absorbance of the extract was recorded at 760 nm after 30 min of incubation at room temperature. Gallic acid was used for plotting standard curve, and TPC was expressed as milligram of gallic acid equivalents (GAE) per gram of the fresh tissue. Antioxidant activity in the tissue extract was estimated using stable diphenyl-1-picrylhydrazyl (DPPH) radical according to the method described by Hatano et al. (1988). Absorbance of the extract was measured at 517 nm after 30 min of incubation at room temperature. The capacity of sample extract to scavenge DPPH radical was calculated using the formula:

$$\text{Scavenging}\;(\%)\;\text{=}\;[({\text{A}}_{\text{0}}\;\text{-}\;{\text{A}}_{\text{1}}){\text{/A}}_{\text{0}})]\;\times \;\text{100};$$

where, A_0_ is the absorbance of the control reaction and A_1_ is the absorbance of the sample. The inhibitory concentration at 50% (extract concentration that cause 50% scavenging of DPPH radical, IC_50_) was determined.

Sodium (Na^+^), potassium (K^+^), calcium (Ca^2+^), and magnesium (Mg^2+^) concentrations in shoot and root tissues were determined according to the method described by Wu et al. (2013) using atomic absorption spectrophotometer. The tissue samples were blot dried followed by determination of FW and DW. Na^+^, K^+^, Ca^2+^, and Mg^2+^ were extracted from the dried plant tissue in 100 mM acetic acid at 90°C for 2 h and cation analysis was performed.

For comprehensive assessment of salt tolerance ability of the wheat genotypes, eight important indicators (biochemical and physiological) were selected. Relative value for the indicator in shoot and root was calculated from treatment (200 mM NaCl) mean values as percentages of the respective mean value under control condition (0 mM NaCl). The relative value for each of these parameters was then standardized according to the formulas given by Bao et al. (2009); one for the indicators which are positively correlated (proline, soluble sugar, TPCs, and antioxidant potential) with salt tolerance [X′_ij_ = (X_ij_ – X_jmin_)/(X_jmax_ – X_jmin_)], and another for the indicators which are negatively correlated (DW, lipid peroxidation, Na^+^/K^+^ ratio, and total chlorophyll content) with salt tolerance [X′_ij_ = 1 - (X_ij_ – X_jmin_)/(X_jmax_ – X_jmin_)]. Herein X′_ij_ = 0, when X_ij_ = X_jmin_; and X′_ij_ = 1, when X_ij_ = X_jmax_. Also X′_ij_ is the standardized value of X_ij_, X_ij_ is the relative value of j indicator for i cultivar, X_jmin_ is the minimum value of the j indicator, X_jmax_ is the maximum value of the j indicator. The relative salt tolerance ability of the wheat genotype was estimated based on the standardized value (SV) for 15 parameters using the formula:

$$\begin{array}{c} \text{Relative}\;\text{salt}\;\text{tolerance}\;\text{=}\;[I_{\left(\text{1}\right)}\;\text{+}\;I_{\left(\text{2}\right)}\;\text{+}\;I_{\left(\text{3}\right)}\;\text{+}\;I_{\left(\text{4}\right)}\;\text{+}\;I_{\left(\text{5}\right)}\;\text{+ }\\ I_{(}{{}_{\text{6}}}_{)}\;\text{+}\;I_{(}{{}_{\text{7}}}_{)}\;\text{+}\;I_{(}{{}_{\text{8}}}_{)}\;\text{+}\;I_{(}{{}_{\text{9}}}_{)}\;\text{+}\;I_{(}{{}_{\text{10}}}_{)}\;\text{+}\;I_{\left(\text{11}\right)}\;\text{+}\;I_{\left(\text{12}\right)}\;\text{+}\;I_{\left(\text{13}\right)}\;\text{+ }I_{\left(\text{14}\right)}\;\text{+}\;I_{\left(\text{15}\right)}]\text{/15}; \end{array}$$

where *I*_(1)_ represents SV of shoot DW, *I*_(2)_ represents the SV of root DW, as specified in **Table 2**.

**Table 2:** Multivariate, comprehensive assessment of salt tolerance level of the wheat genotypes based on relative values of physiological and biochemical indicators/parameters at 200 mM of NaCl stress.

Parameter/Indicator	Standardized value^∗^			
	KRL-210	Kharchia-65	HD-2329	WH-542
Dry weight of shoot, *I*_(1)_	0.603	1	0	0.344
Dry weight of root, *I*_(2)_	1	0.728	0	0.169
Proline content in shoot, *I*_(3)_	0.813	1	0	0.221
Proline content in root, *I*_(4)_	0.721	1	0	0.303
Soluble sugar content in shoot, *I*_(5)_	0.653	1	0	0.197
Soluble sugar content in root, *I*_(6)_	0.817	1	0	0.106
Total phenolics content in shoot, *I*_(7)_	0.509	1	0	0.184
Total phenolics content in root, *I*_(8)_	0.504	1	0	0.181
Lipid peroxidation in shoot, *I*_(9)_	0.838	1	0	0.212
Lipid peroxidation in root, *I*_(10)_	0.758	1	0	0.103
Antioxidant potential of shoot, *I*_(11)_	0.628	1	0	0.396
Antioxidant potential of root, *I*_(12)_	0.726	1	0	0.489
Na^+^/K^+^ ratio in shoot, *I*_(13)_	0.805	1	0	0.309
Na^+^/K^+^ ratio in root, *I*_(14)_	0.964	1	0	0.316
Total chlorophyll content of leaf, *I*_(15)_	0.859	1	0	0.137
Mean	0.746	0.982	0.000	0.244
Rank	2nd	1st	4th	3rd

*^∗^Calculated from the respective relative values.*

Cloning and sequencing of the *HKT* (*HKT1;4, HKT2;1*, and *HKT2;3*) genes from the contrasting wheat (Kharchia-65 and HD-2329) genotypes have been described earlier (Singh et al., 2015; Kumar et al., 2017). The partial coding sequences (KR262818, KR262819; HF937364, HG934157; HF937363, KR018843) of the *HKTs* cloned from the contrasting wheat genotypes were analyzed using bioinformatics tools. For comparison of the nucleotide and amino acid sequences, to analyze nucleotide deletions/insertions, transitions/transversions and amino acid substitution, the sequences were aligned using ClustalX software (Thompson et al., 1997) and visualized using BioEdit (Hall, 1999) graphical view (Srivastava et al., 2011). Target-template alignment was used for the selection of template and the template with the highest quality was selected for model building using ProMod3^1^ (Benkert et al., 2011). Homology modeling was performed using the BLAST (Altschul et al., 1997) against the SWISS-MODEL template library (SMTL). For modeling of protein structure to predict 3D model and to perform comparative analysis, a fully automated SWISS-MODEL servers^2^ was used. Conserved domain analysis was performed using the NCBI Conserved Domains Database^3^ (Marchler-Bauer et al., 2015) to annotate the location of evolutionarily conserved domain footprints, and functional sites inferred from the footprints. To study expression of *HKTs* in shoot and root of the wheat genotypes, RT-qPCR was performed following the MIQE guidelines (Bustin et al., 2009) as described earlier (Singh et al., 2015). Actin was used as reference/housekeeping gene.

To investigate the role of epigenetic variation in regulation of *HKTs* expression, bisulfite sequencing of the genes was carried out using the modified genomic DNA from the most contrasting wheat (Kharchia-65 and HD-2329) genotypes for PCR amplification using ExTaq DNA polymerase and the gene-specific primers as mentioned elsewhere (Kumar et al., 2017). The PCR products were cloned in pGEM–T Easy Vector and 10 independent clones of each sample were outsourced for sequencing. Methylation data were analyzed using the Kismeth software, and differentially methylated regions (DMRs) were identified in every 100-bp window with a step size of 50 bp by comparing bisulfite sequences for different genotypes, tissues, and treatments using the Fisher exact test with a *P*-value cut-off of 0.05 (Wang et al., 2016).

### Statistical Analysis

The data were analyzed using statistical software (SPSS 19.0). Fisher’s least significant difference (LSD) was performed to determine significant difference between means at a significance level of *P* ≤ 0.05 and reported as the mean ± standard deviation (SD).

## Results

### Morphological Responses of Wheat Genotypes under Salt Stress

**FIGURE 1:**
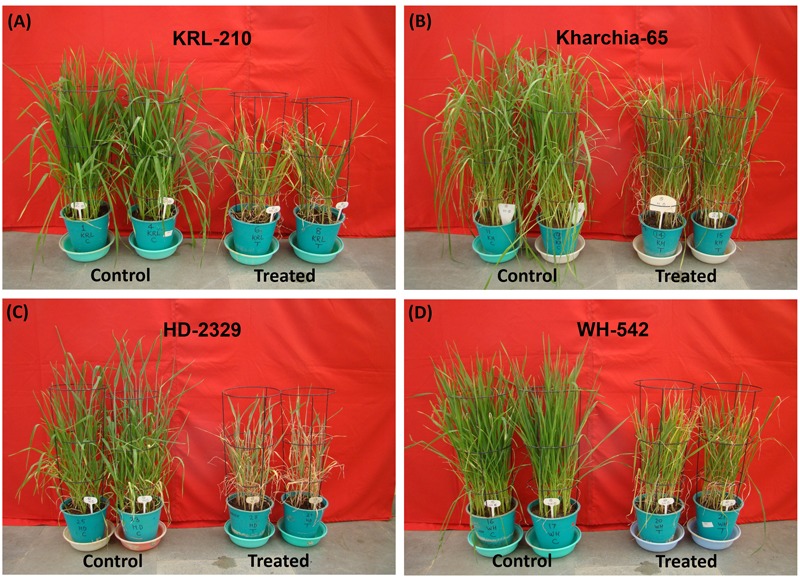
Effects of salt stress on growth and development of bread wheat genotypes: Kharchia-65 control vs. salt treated **(A)**, KRL-210 control vs. salt treated **(B)**, HD-2329 control vs. salt treated **(C)** and WH-542 control vs. salt treated **(D)**. Two-week-old seedlings were treated with 200 mM NaCl for 14 days.

Preliminary experimentation on seedlings of randomly selected bread wheat genotypes with varying NaCl concentrations (0, 50, 100, 150, 200, 250, and 300 mM) for different durations (0, 3, 6, 9, 12, 14, and 16 days) indicated that >200 mM NaCl stress for >14 days was lethal, particularly for HD-2329 (Supplementary Table S1). Morphological, biochemical, and physiological responses of the wheat genotypes showed that stress treatment with 200 mM NaCl for 14 days was most appropriate for studies on stress responses of wheat genotypes associated with physio-biochemical traits and defense pathways (**Figure 1** and Supplementary Table S1). Therefore, comparative analyses of the salt-modulated various changes occurring in the wheat genotypes and assessment of their ability to acclimatization under the stress was carried out by imposing the stress with 200 mM NaCl for 14 days only. Morphological attributes of the wheat genotypes grown under control and stress conditions indicated noticeable effects of the stress on growth and development of all the four wheat genotypes. The stress showed significant effects on plant height, number of tillers and leaf senescence of the wheat plant (**Figure 1** and **Table 1**).

### Physiological Responses of Wheat Genotypes under Salt Stress

**FIGURE 2:**
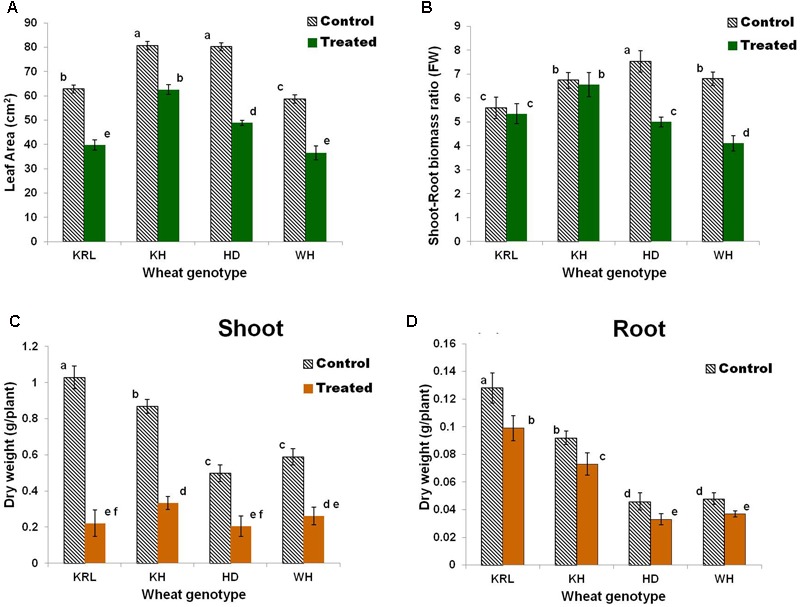
Effects of salt stress on bread wheat genotypes. Leaf-area **(A)**, shoot-root biomass ratio **(B)**, dry weight of shoot **(C)**, and dry weight of root **(D)**. Two-week-old seedlings were treated with 200 mM NaCl for 14 days. KRL = KRL-210, KH = Kharchia-65, HD = HD-2329, and WH = WH-542. Bars indicate ± SD. Columns with different lower case letters indicate significant difference at *P* < 0.05 [Fisher’s least significant difference (LSD) test].

Comparative evaluation of the wheat genotypes under control and salt stress conditions indicated that the salt stress caused significant reduction in leaf-area in all the genotypes (**Figure 2A**). Comparison based on shoot-root biomass ratio indicated no significant change in case of Kharchia-65 and KRL-210 on salt stress imposition, while considerable reduction (33-42%) was observed in case of WH-542 and HD-2329 (**Figure 2B**). Drastic reduction (56-78%) in shoot dry matter of the wheat genotypes was observed due to the stress (**Figure 2C**), while reduction in root dry matter (20-28%) was comparatively less (**Figure 2D**).

**FIGURE 3:**
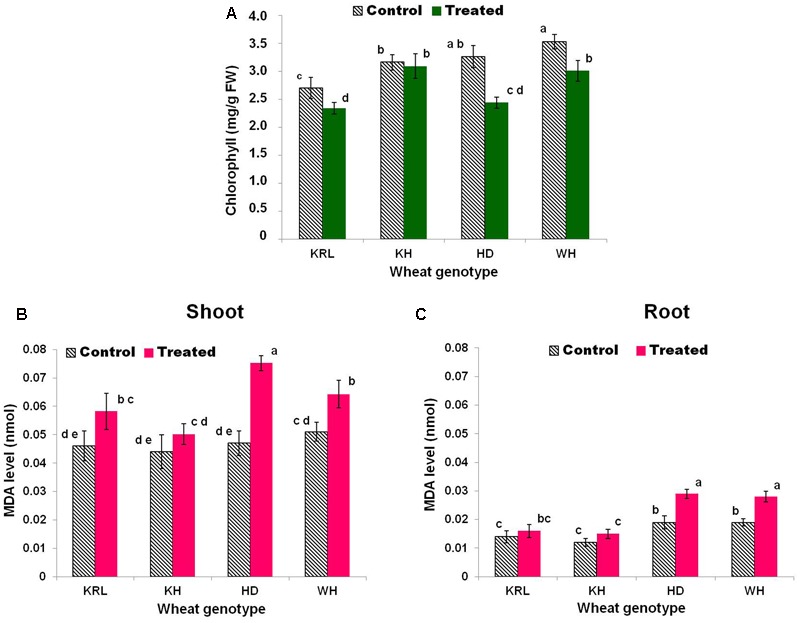
Effects of salt stress on total chlorophyll content **(A)**, malondialdehyde (MDA) level (lipid peroxidation) in shoot **(B)**, and MDA level in root **(C)** in the wheat genotypes. Two-week-old seedlings were treated with 200 mM NaCl for 14 days. KRL = KRL-210, KH = Kharchia-65, HD = HD-2329, and WH = WH-542. Bars indicate ± SD. Columns with different lower case letters indicate significant difference at *P* < 0.05 (Fisher’s LSD test).

Salt stress caused only a minor/insignificant effect on chlorophyll content in Kharchia-65, while KRL-210 and WH-542 showed significant reduction (15 and 19%, respectively) in chlorophyll content. The maximum (>25%) reduction in total chlorophyll content due to the salt stress was observed in HD-2329 (**Figure 3A**).

### Malondialdehyde and Membrane Stability

Estimation of lipid peroxidation (in terms of MDA level) in shoot tissues of the wheat genotypes under salt stress revealed that increase in lipid peroxidation (MDA level) was maximum (60%) in HD-2329 compared to a minor, insignificant increase in case of Kharchia-65 and KRL-210 (**Figures 3B,C**). The increase in lipid peroxidation in root tissues was observed to be non-significant in case of Kharchia-65 and KRL-210, but significantly higher in case of WH-542 and HD-2329. Besides, the level of lipid peroxidation under control condition and the increase due to salt stress were more prominent in shoot compared to that in root.

The MSI of the wheat genotypes under the stress was assessed by measuring electrical conductivity/electrolyte leakage. MSI of shoot was observed to be better than that of root in all the four genotypes. The maximum reduction (25%) in MSI was observed in root of HD-2329, while the minimum reduction was observed in shoot of Kharchia-65 (11%). Although MDA level in shoot was observed to be significantly higher, MSI was found to be maintained high in shoot compared to that observed in root (Supplementary Table S2).

### Osmolyte Concentration

**FIGURE 4:**
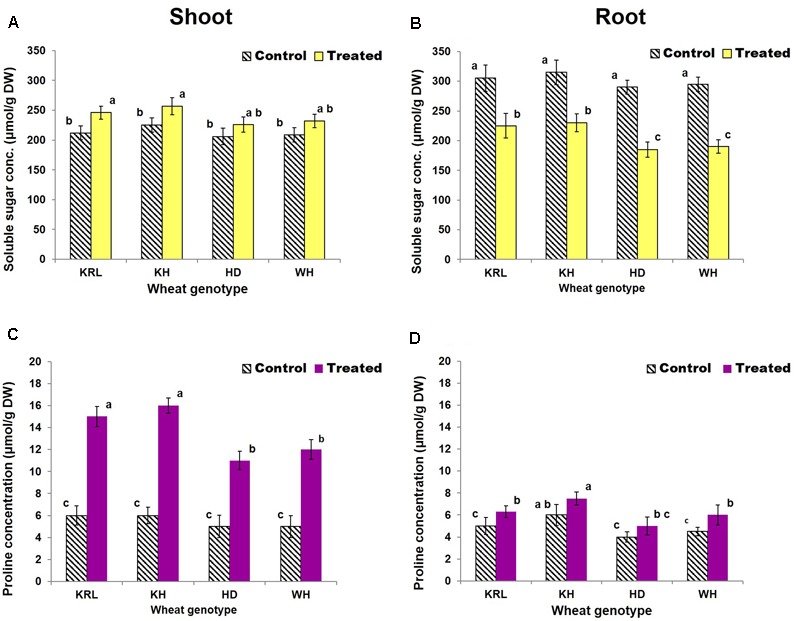
Effects of salt stress on soluble sugar content in shoot **(A)**, soluble sugar content in root **(B)**, proline content in shoot **(C)**, and proline content in root **(D)**. Two-week-old seedlings were treated with 200 mM NaCl for 14 days. KRL = KRL-210, KH = Kharchia-65, HD = HD-2329, and WH = WH-542. Bars indicate ± SD. Columns with different lower case letters indicate significant difference at *P* < 0.05 (Fisher’s LSD test).

Total soluble sugar content of root was observed to be higher than that of shoot under control condition. Salt stress caused significant increase in TSS in shoot of KRL-210 and Kharchia-65 (**Figure 4A**). By contrast, the TSS was observed to decrease significantly (26-36%) under the stress in all the four genotypes with maximum reduction in HD-2329 and minimum in Kharchia-65 (**Figure 4B**).

With a minor variation, the proline content of shoot and root of the wheat genotypes was similar under control condition. Salt stress caused a considerable increase in proline content (120-166%) in shoot of all the four genotypes. The maximum increase was observed in Kharchia-65, while the minimum was observed in HD-2329. By contrast, only a minor/non-significant increase was observed in root of all the four wheat genotypes due to the salt stress (**Figures 4C,D**).

### Phytophenolics Content

**FIGURE 5:**
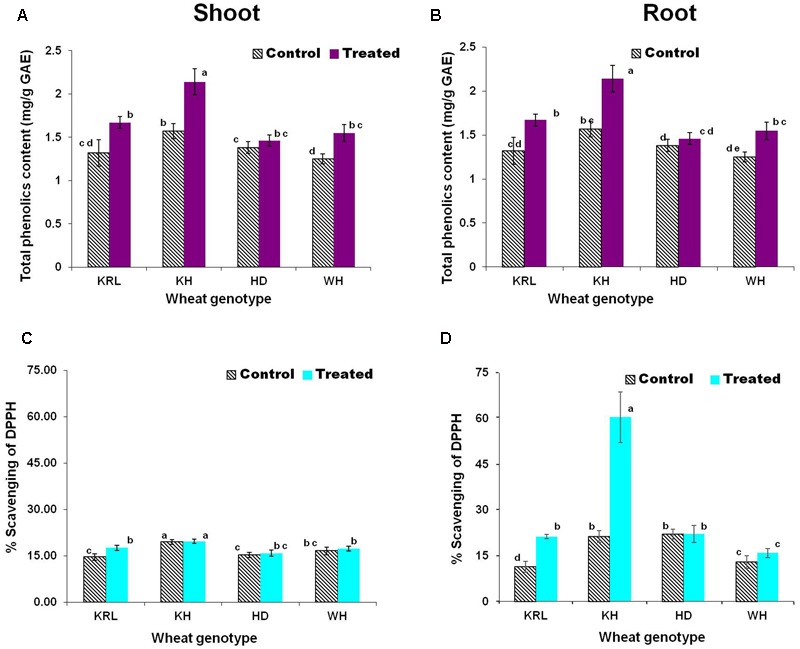
Effects of salt stress on total phenolics content (TPC) in shoot **(A)**, TPC in root **(B)** expressed as mg/g gallic acid equivalents (GAE), antioxidant activity in shoot **(C)** and antioxidant activity in root **(D)**. Two-week-old seedlings were treated with 200 mM NaCl for 14 days. KRL = KRL-210, KH = Kharchia-65, HD = HD-2329, and WH = WH-542. Bars indicate ± SD. Columns with different lower case letters indicate significant difference at *P* < 0.05 (Fisher’s LSD test).

To examine the effect of salt stress on accumulation of phytophenols (secondary metabolites) in the wheat genotypes, comparative analysis of TPC was performed. The analysis revealed considerable variation in TPC in the wheat genotypes. Though a similar trend was observed in shoot and root tissues, the stress triggered significant increase in TPC in Kharchia-65, KRL-210 and WH-542, except for HD-2329. The maximum increase (∼35%) in TPC was observed in shoot and root of Kharchia-65. The increase was non-significant in both shoot and root of HD-2329 (**Figures 5A,B**).

### Antioxidant Activity

The stress modulated antioxidant activity in shoot and root of the wheat genotypes. While significant increase in antioxidant activity (% scavenging of DPPH) in shoot and root of KRL-210 was observed due to salt stress, it was constitutively higher in case of Kharchia-65 (**Figures 5C,D**). Only minor/non-significant increase in antioxidant activity was observed in shoot and root of WH-542 and HD-2329 under the stress. Comparison of TPC and antioxidant activity (% scavenging of DPPH) indicated that TPC do contribute in antioxidant potential but other antioxidants also play role in protecting the plants under salt stress. Interestingly, antioxidant activity in shoot and root of the wheat genotypes was observed to be of the same level.

### Relative Distribution of Na^+^, K^+^, Ca^2+^ and Mg^2+^ in Shoot and Root

**FIGURE 6:**
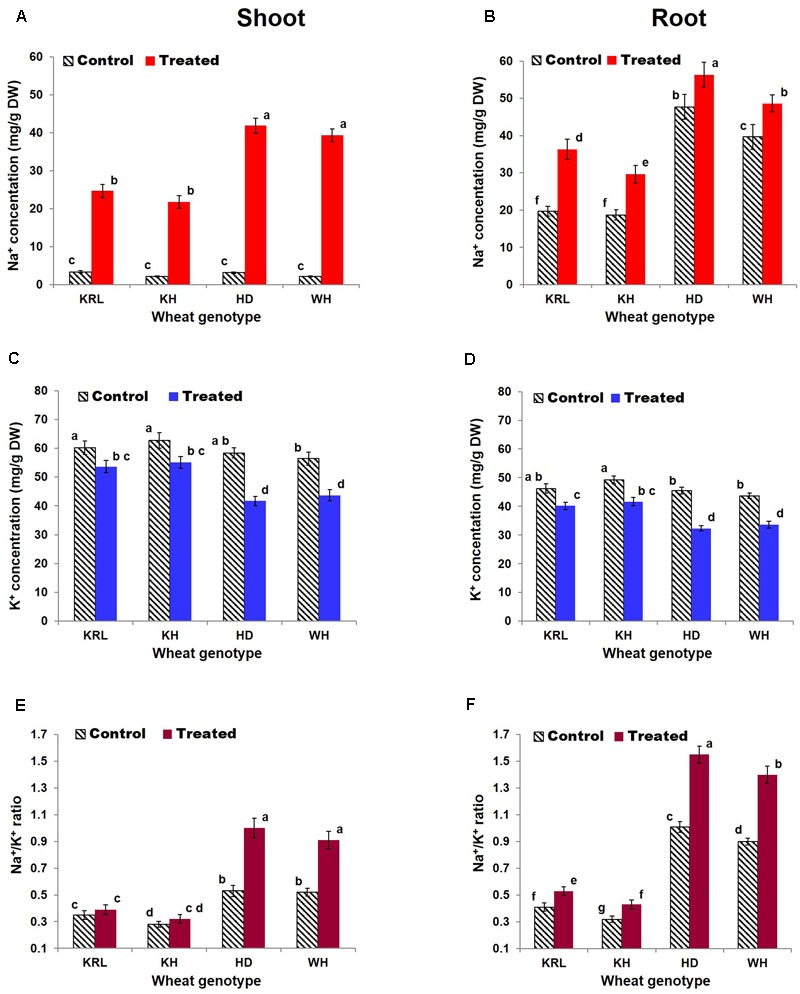
Effects of salt stress on Na^+^ concentration in shoot **(A)**, Na^+^ concentration in root **(B)**, K^+^ concentration in shoot **(C)**, K^+^ concentration in root **(D)**, Na^+^/K^+^ ratio in shoot **(E)**, and Na^+^/K^+^ ratio in root **(F)**. Two-week-old seedlings were treated with 200 mM NaCl for 14 days. KRL = KRL-210, KH = Kharchia-65, HD = HD-2329, and WH = WH-542. Bars indicate ± SD. Columns with different lower case letters indicate significant difference at *P* < 0.05 (Fisher’s LSD test).

Comparison of Na^+^ distribution between shoot and root tissues under control condition revealed accumulation of Na^+^ in higher concentration in root, indicating that root is the primary accumulator of Na^+^. However, under salt stress significant amount of Na^+^ got transported to the shoot (**Figures 6A,B**). Na^+^ concentration in shoot under the stress increased considerably in HD-2329 and WH-542 (10- to13-fold). The increase was comparatively less (6-fold) in Kharchia-65 and KRL-210 (**Figures 6A,B**). Root of Kharchia-65 and KRL-210 absorbed comparatively lesser amount of Na^+^ compared to that by HD-2329 and WH-542.

Inhibitory effect of the stress was observed on absorption/distribution of K^+^, with a significant (10-30%) reduction in K^+^ concentration in shoot and root of the wheat genotypes. K^+^ concentration was observed to be higher in shoot of all the genotypes compared to that in root (**Figures 6C,D**). Although 10-30% reduction in K^+^ concentration was observed in shoot and root of the wheat genotypes, it was maintained at higher level in Kharchia-65 and KRL-210 compared to that in HD-2329 and WH-542. Due to the stress, a significant increase in Na^+^/K^+^ ratio was observed in shoot and root of all the tested genotypes with a more prominent (up to 80%) increase in shoot (**Figures 6E,F**). Similar to the K^+^ concentration, Ca^2+^ also decreased significantly in shoot and root of the wheat genotypes tested (Supplementary Table S3). The stress showed significant effects on absorption/distribution of Na^+^, K^+^, and Ca^2+^ in root and shoot, but it did not affect absorption/transport of Mg^2+^ (Supplementary Table S3). Interestingly, Mg^2+^ concentration was found to be comparable in shoot and root tissues.

### Comparison of Wheat Genotypes

Multivariate, comprehensive analysis of four selected wheat genotypes based on the SVs (calculated from the respective relative values) for each of the 8 variables/indicators (15 parameters) ranked the wheat genotypes according to their relative salt tolerance ability (**Table 2**). The SV of 0 for a parameter indicates the least relative value observed for that parameter, and the SV of 1 for a parameter indicates the highest relative value observed for that parameter. Comprehensive assessment of the wheat genotypes ranked HD-2329 at fourth position indicating it to be the most salt-sensitive. Kharchia-65 was ranked first with the SV of 1 for most (14 out of 15) of the parameters. KRL-210 and WH-542 were ranked second and third, respectively, with considerable (3-fold) difference in their mean SV (**Table 2**).

### Salt-Modulated Accumulation of Transcripts for the Ion-Transporters

**FIGURE 7:**
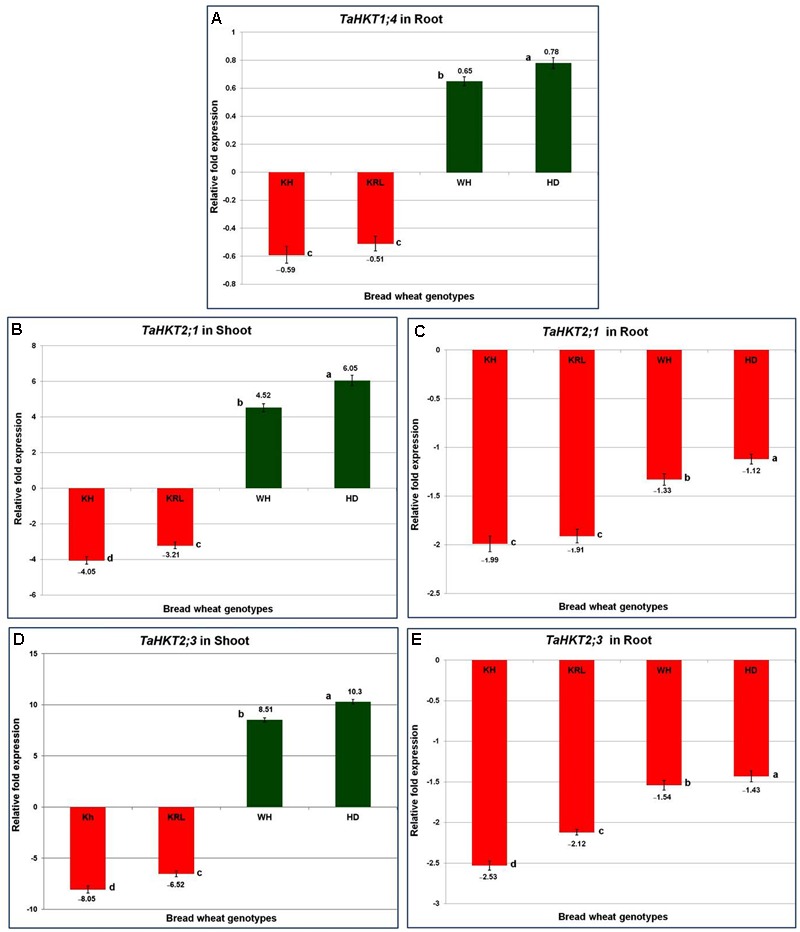
Quantitative expression (RT-qPCR) analysis of salt-responsive high-affinity potassium transporter (*HKT*) genes. Transcript accumulation of *TaHKT1;4* in root **(A)**, *TaHKT2;1* in shoot **(B)**, *TaHKT2;1* in root **(C)**, *TaHKT2;3* in shoot **(D)**, and *TaHKT2;3* in root **(E)**. Two-week-old seedlings were treated with 200 mM NaCl for 14 days. KRL = KRL-210, KH = Kharchia-65, HD = HD-2329, and WH = WH-542. The results are mean fold change in relative expression over the control with three biological and three technical replicates, normalized with actin (reference) gene expression. Bars represent standard deviation. Columns with different lower case letters indicate significant difference at *P* < 0.05 (Fisher’s LSD test).

To investigate the effect of salt stress on transcription of *HKT*s and to correlate it with salt tolerance ability of the wheat genotypes, relative expression of three *HKTs* was examined. One of the candidate genes (*TaHKT1;4*) shows root-specific expression with a well-defined functions (Kumar et al., 2017), while the other two genes (*TaHKT2;1* and *TaHKT2;3*) express in both shoot and root (Singh et al., 2015). Quantitative expression analysis of *TaHKT1;4* indicated that the gene was downregulated by the stress in root of Kharchia-65 and KRL-210. By contrast, the gene was observed to be upregulated in HD-2329 and WH-542 (**Figure 7A**). *TaHKT2;1* and *TaHKT2;3* genes were found to be differentially expressed in shoot and root tissues. Expression analysis of *TaHKT2;1* revealed that it was downregulated in shoot of Kharchia-65 (4.05-fold) and KRL-210 (3.21-fold), but it was upregulated in WH-542 (4.52-fold) and HD-2329 (6.05-fold) under the stress (**Figure 7B**). In root, the gene was observed to be downregulated under the stress in all the four genotypes (**Figure 7C**). Expression analysis of *TaHKT2;3* showed a similar pattern with that of *TaHKT2;1*. The gene was observed to be downregulated in shoot of Kharchia-65 (8.05-fold) and KRL-210 (6.52-fold) but upregulated in WH-542 (8.51-fold) and HD-2329 (10.30-fold) (**Figure 7D**). The gene was found to be downregulated (maximum 2.5-fold in Kharchia-65) in root of all genotypes under the stress (**Figure 7E**).

### Genetic Variations in the *HKT* Genes

**FIGURE 8:**
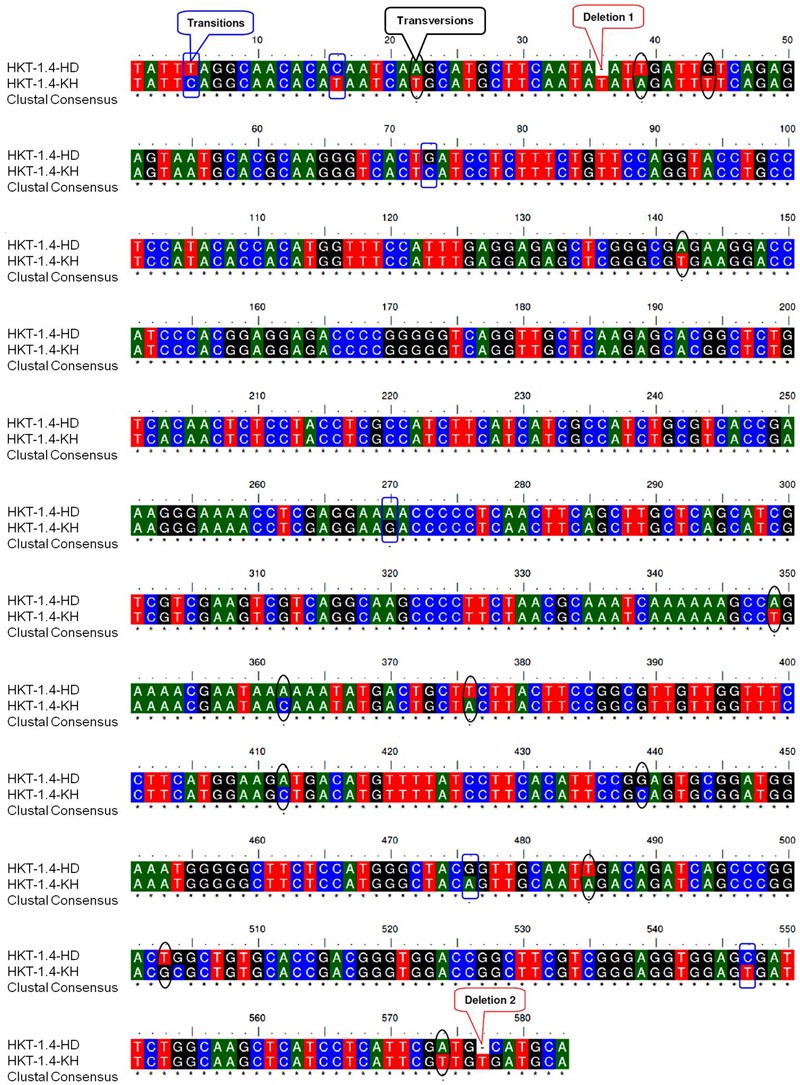
Alignment of *HKT1;4* sequences from HD-2329 (HD) and Kharchia-65 (KH) showing point mutations (deletions, transitions, and transversions).

**FIGURE 9:**
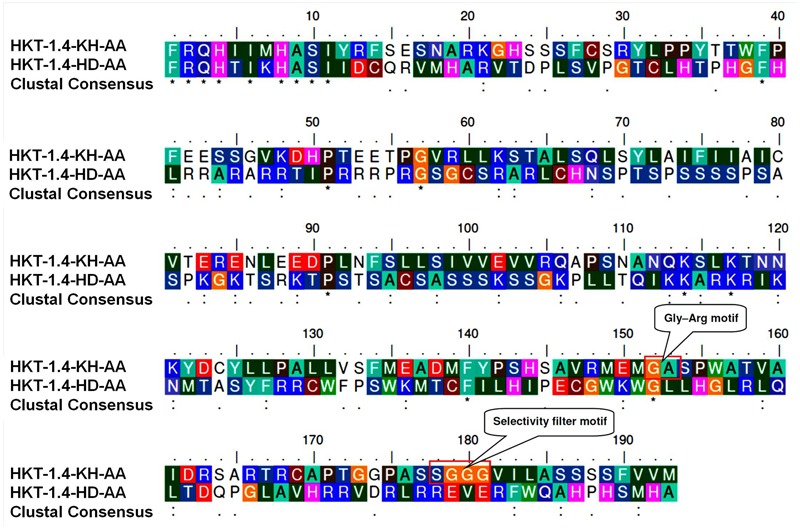
Alignment of amino acid sequences of TaHKT1;4.2 (HKT-1.4-KH-AA) and TaHKT1;4.3 (HKT-1.4-KH-AA) depicting amino acid substitutions due to point mutatuions, and locations (presence/absence) of certain conserved functional motifs.

**FIGURE 10:**
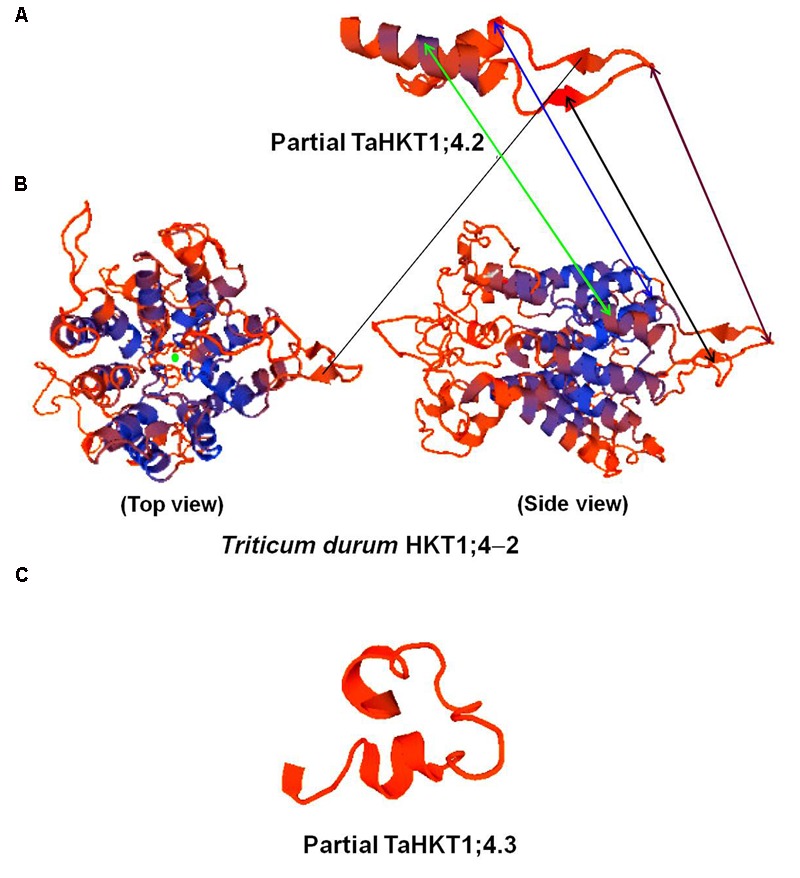
SWISS-MODEL of the partial TaHKT1;4.2 **(A)**, TdHKT1;4-2 **(B)**, and partial TaHKT1;4.3 **(C)** built with the ProMod3 software.

**FIGURE 11:**
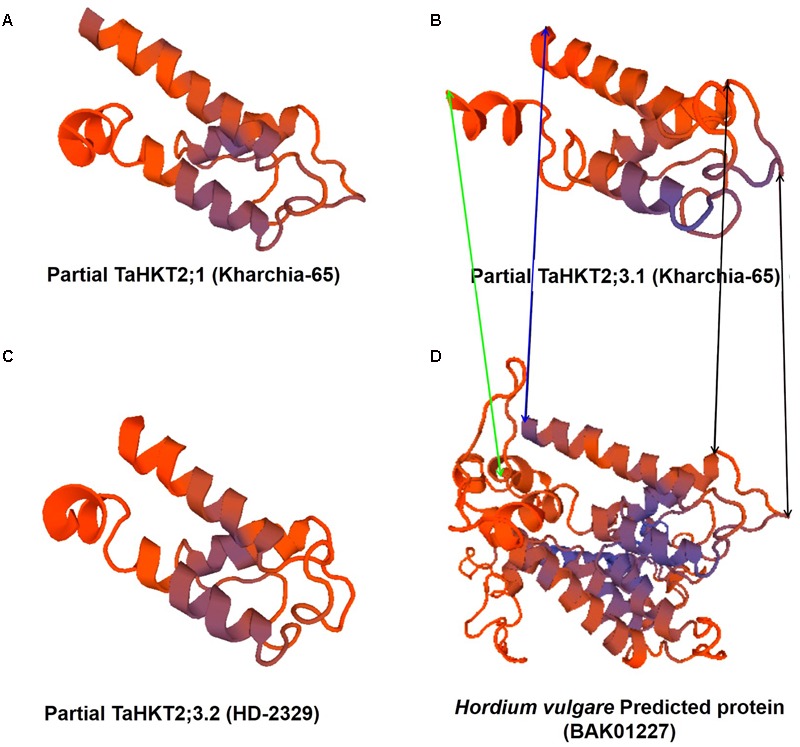
SWISS-MODEL of the partial TaHKT2;1 **(A)**, TaHKT2.3.1 **(B)**, TaHKT2;3.2 **(C)** and a predicted protein (BAK01227) of *Hordeum vulgare* subsp. *vulgare* **(D)** built with the ProMod3 software.

Alignment of the cloned partial sequences of *HKT1;4* gene (KR262818, KR262819) from the most contrasting salt-responsive genotypes (Kharchia-65 and HD-2329) indicated that the gene contains 2 deletions, 6 transitions, and 12 transvertions in HD-2329 (**Figure 8**). The point mutations observed in *TaHKT1;4.3* (*TaHKT1;4* from HD-2329) appeared to have significant effect on its amino acid sequence (**Figure 9**), structure (**Figure 10**) and function. Due to the point mutations “Gly-Arg motif” and “selectivity filter motif” (Ser-Gly-Gly-Gly) disappeared from TaHKT1;4.3 (TaHKT1;4 in HD-2329) but they were present in TaHKT1;4.2 (Kharchia-65) (**Figure 9**). Alignment of amino acid sequences of TaHKT2;3.1 (Kharchia-65) with TaHKT2;3.2 (HD-2329) showed four substitutions. At the first place, Isoleucine was substituted by Valine, at the second position the Valine was substituted by Alanine, at the third position substitution was from Serine to Threonine, and at fourth position Valine was substituted by Isoleucine. However, no detectable effects of the mutations could be seen on the structure of the protein (**Figure 11**).

Homology modeling of HKT1;4 revealed that TaHKT1;4.2 possesses maximum (21.95%) sequence identity with Ktr potassium uptake protein of bacteria. By contrast, TaHKT1;4.3 did not show homology with any of the Na^+^/K^+^ transporter, but significant (43.33%) sequence identity with ATP synthase β chain mitochondrial precursor. SWISS-MODEL of the TaHKT1;4 proteins built with ProMod3 clearly showed that the partial TaHKT1;4.2 protein resembled with a part of the model predicted for TdHKT1;4-2 (HKT1;4 from *Triticum durum*), but TaHKT1;4.3 showed an entirely different model (**Figure 10**). However, conserved domains analysis for the TaHKT1;4.2 and TaHKT1;4.3 revealed presence of a 2a38euk domain specific to the potassium uptake proteins of Trk family from Gram-positive and Gram-negative bacteria, yeast and wheat.

Homology model of HKT2;1.1 (Kharchia-65) revealed that it possesses 18-23% sequence identity with Ktr system potassium uptake protein B, Voltage-gated sodium and potassium channels. Conserved domain analysis of the partial TaHKT2;1.1 revealed that it contains 2a38euk domain of Trk family potassium uptake proteins from Gram-positive and Gram-negative bacteria, yeast and wheat. SWISS-MODEL of the partial TaHKT2;1 clearly showed that the partial protein resembled with a part of the model predicted for *Hordeum vulgare* subsp. *vulgare* (BAK01227) protein (**Figures 11A,D**). Homology modeling of TaHKT2;3.1 (Kharchia-65) showed that it possesses 26.83% sequence identity with Ktr potassium uptake protein B, while TaHKT2;3.2 (HD-2329) showed 23.75% sequence identity with the Ktr potassium uptake protein B. Conserved domain analyses for the partial TaHKT2;3.1 and TaHKT2;3.2 showed that they contain 2a38euk domain of Trk family K^+^ uptake proteins. SWISS-MODEL of the partial TaHKT2;3.1 and TaHKT2;3.2 clearly showed that the proteins resembled with part of the model predicted for *Hordeum vulgare* subsp. *vulgare* (BAK01227) protein (**Figures 11B–D**).

### Epigenetic Changes in the *HKTs*

**FIGURE 12:**
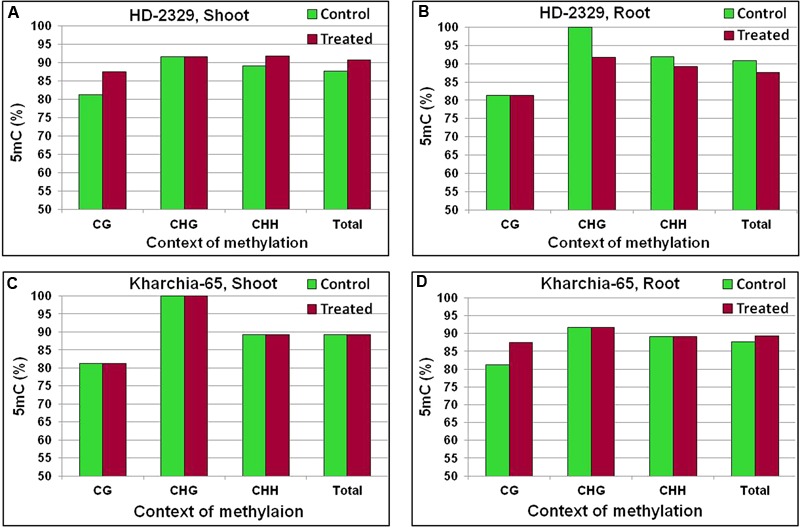
Cytosine methylation [5-methylcytosine (5mC)] in *TaHKT1;4* in different contexts (CG, CHG, and CHH) in contrasting wheat genotypes HD-2329 and Kharchia-65 under control and 200 mM NaCl stress. 5mC in shoot of HD-2329 (salt-sensitive genotype) **(A)**, 5mC in root of HD-2329 **(B)**, 5mC in shoot of Kharchia-65 (salt-tolerant genotype) **(C)**, 5mC in root of HD-2329 **(D)** as estimated using bisulfite sequencing.

A minor variation in 5-methylcytosine (5mC) content was observed in the coding region of *TaHKT1;4* (Kumar et al., 2017). The last quarter of the gene body, used for quantitative analysis of 5mC, was observed to contain context-specific variation in 5mC (**Figure 12**). Increase in 5mC content in CG and CHH contexts was observed in the shoot of HD-2329 under salt stress. By contrast, no increase in 5mC was observed in Kharchia-65 (**Figures 12A,C**). A decrease in 5mC content in CHG and CHH contexts was observed in root of HD-2329 under salt stress (**Figure 12B**). But, increase in 5mC content was observed in CG context in root of Kharchia-65 (**Figure 12D**). However, no considerable variation was observed either in cytosine methylation or in DMR in *TaHKT1;4*.

**FIGURE 13:**
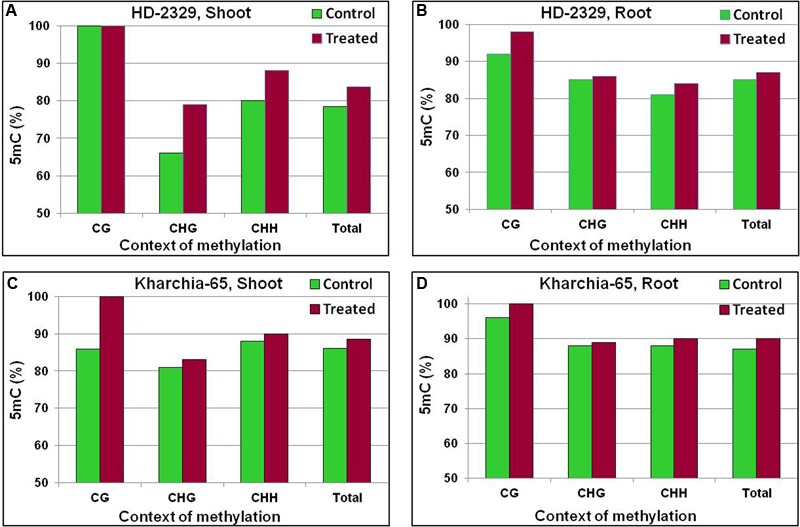
Cytosine methylation (5mC) in *TaHKT2;1* at different contexts (CG, CHG, and CHH) in the contrasting wheat genotypes Kharchia-65 and HD-2329 under control and 200 mM NaCl stress conditions. 5mC in shoot of HD-2329 (salt-sensitive genotype) **(A)**, 5mC in root of HD-2329 **(B)**, 5mC in shoot of Kharchia-65 (salt-tolerant genotype) **(C)**, and 5mC in root of HD-2329 **(D)** as estimated using bisulfite sequencing.

The coding region of TaHKT2;1 (second quarter of the gene body) used for quantitative analysis of 5mC was found to contain variations in 5mC content with respect to the genotypes, tissues, and salt stress (**Figure 13**). The basic methylation level (under control) in shoot of the salt-tolerant genotype was found to be ∼12% higher than that in the salt-sensitive genotype. Salt stress further increased the methylation level in these contexts. With all the cytosines methylated in the CG context, an increase in 5mC was observed in CHG and CHH contexts in shoot of HD-2329 under salt stress (**Figure 13A**). Increase in 5mC content was observed in Kharchia-65 in all the three contexts under salt stress, but the maximum percent increase was observed in the CG context (**Figure 13C**). Similarly, increase in 5mC content was observed in all three contexts in the root of both the genotypes under salt stress (**Figures 13B,D**), but the total methylation was higher in case of Kharchia-65.

**FIGURE 14:**
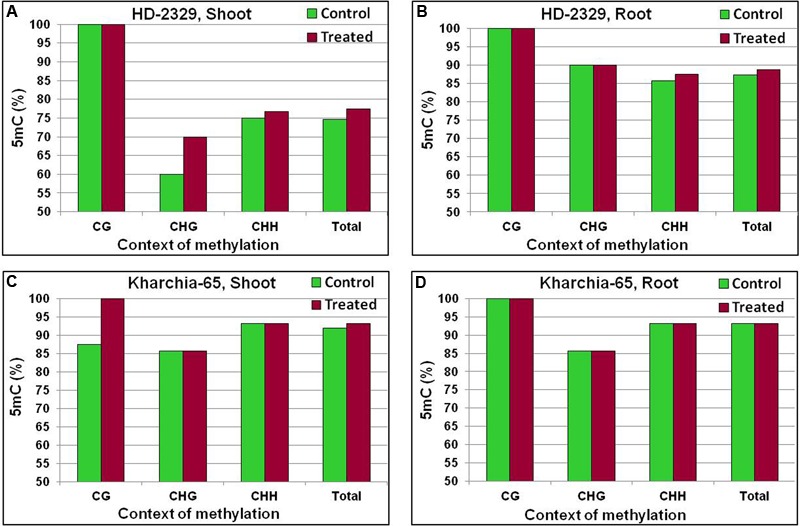
Cytosine methylation (5mC) in *TaHKT2;3* at different contexts (CG, CHG, and CHH) in the contrasting wheat genotypes Kharchia-65 and HD-2329 under control and 200 mM NaCl stress conditions. 5mC in shoot of HD-2329 (salt-sensitive genotype) **(A)**, 5mC in root of HD-2329 **(B)**, 5mC in shoot of Kharchia-65 (salt-tolerant genotype) **(C)**, and 5mC in root of HD-2329 **(D)** as estimated using bisulfite sequencing.

Coding region of the TaHKT2;3 (first quarter of the gene body) used for the quantitative analysis of 5mC showed variations in 5mC content with respect to the genotypes, tissues, and salt treatments (**Figure 14**). With all the cytosines methylated in the CG context, an increase in 5mC content was observed in CHG and CHH contexts in shoot of HD-2329 under salt stress (**Figure 14A**). Only a minor increase in 5mC content was observed in CHH context in the root of HD-2329 under salt stress (**Figure 14B**). Although increase in 5mC content was observed in CG context only in shoot of Kharchia-65, the total methylation (94%) was considerably higher than that of HD-2329 (78%) (**Figure 14C**). With the basic methylation level considerably higher (∼94%), no further increase in 5mC content was observed in root of Kharchia-65 (**Figure 14D**). Thus, a significant variation in 5mC and DMR was observed in TaHKT2;1 and TaHKT2;3 (Supplementary Figure S1).

## Discussion

To study salt stress responses of the wheat genotypes, we first optimized the concentration and duration of salt (NaCl) stress treatment using a pair of randomly selected, locally available contrasting wheat genotypes and a few indicative biochemical/physiological traits/parameters. Results of the preliminary experiment indicated that stress imposition with >200 mM NaCl for >14 days caused irreversible damage to the plants, particularly to the sensitive genotype (Supplementary Table S1). Therefore, subsequent experiments were carried out with 200 mM NaCl for 14 days to study the various changes occurring initially the plants as adaptive measures, and to assess their potential of acclimatization under the stress. Morphological symptoms, biochemical and physiological responses of the wheat genotypes revealed that stress treatment with 200 mM NaCl for 14 days was most appropriate for comparative evaluation of the wheat genotypes (**Figure 1** and Supplementary Table S1). The available literature corroborated our finding as we found that 200 mM NaCl has been commonly used for evaluation of salt tolerance of different plant species including wheat (Tammam et al., 2008; Bao et al., 2009; Wu et al., 2013). Hence, salt treatment with 200 mM NaCl for 14 days was used in the subsequent experiments.

Kharchia-65 (Sairam et al., 2002; Singh et al., 2015) and KRL-210 (Kumar et al., 2015) were reported earlier to be salt-tolerant bread wheat genotypes; however, their relative tolerance level has not been known. Stress imposition for 14 days followed by observations on plant height, number of tillers and leaf senescence (**Figure 1** and **Table 1**) provided an indication about salt tolerance ability of the wheat genotypes. However, the result was subjected to verification based on certain physio-biochemical traits. Changes in leaf-area, shoot-root biomass ratio and dry matter yield due to the stress corroborated the above observations on salt tolerance ability of the wheat genotypes. Accumulation of excessive salts in photosynthetic tissues has been reported earlier to cause decrease in leaf-area and senescence of leaf (Rauf et al., 2010). Effects of salt stress on plant biomass have been suggested to be an important criteria for evaluating salt tolerance in crop plants (Bao et al., 2009). A significant reduction in the biomass ratio was observed in HD-2329 and WH-542 (**Figure 2**).

No significant reduction in total chlorophyll content in Kharchia-65, but significant reduction in the other genotypes, supported better salt tolerance ability of Kharchia-65. By contrast, with maximum reduction in total chlorophyll content of HD-2329 the genotype emerged as the most salt-sensitive (**Figure 3A**). The observed effect of salt stress on chlorophyll content is in agreement with those reported earlier in kidney bean, cabbage, and different species of wheat (Rauf et al., 2010). Accumulation of Na^+^ has been reported to adversely affect chlorophyll biosynthesis and photosynthesis process (particularly photosystem-II) in plants (Jiang et al., 2017).

Reactive oxygen species has been considered as a central component of plant’s adaptive responses to biotic and abiotic stresses. Production of ROS is intimately related with the stressful conditions which leads to the membrane damage and electrolyte leakage (Kumar et al., 2015). Therefore, lipid peroxidation and MSI were used to assess salt tolerance ability of the different genotypes (Supplementary Tables S1, S2). The maximum increase in MDA level/lipid peroxidation was observed in HD-2329, while the minimum increase was observed in Kharchia-65 (**Figures 3B,C**). With a non-significant increase in MDA level in shoot and root under the stress, Kharchia-65 could minimize electrolyte leakage and showed higher MSI (Supplementary Table S2). With a significant increase in MDA level (negatively correlated with stress tolerance), HD-2329 showed maximum reduction in MSI. These results are in agreement with the findings of Rao et al. (2013) who reported lower lipid peroxidation and better membrane stability in salt-tolerant genotypes of different plant species.

The compatible solutes, particularly soluble sugars and proline, play significant role in osmotic adjustment/structural stability during abiotic stress (Romero-Aranda et al., 2006). In fact, cellular machinery releases compatible solutes to maintain redox potential under the stress. We observed mobilization of TSS from root to shoot under the stress (**Figures 4A,B**). Increased accumulation of soluble sugar (in shoot) and proline (**Figure 4**) was in agreement with the earlier observations of Romero-Aranda et al. (2006). Intriguingly, a significant reduction in soluble sugar was observed in root of the wheat genotypes which substantiate the observation of Wu et al. (2013). The increased concentration of compatible solutes might be one of the key factors in alleviating the stress either via osmotic adjustment or by conferring desiccation resistance. Soluble sugar and proline have been suggested to enhance salt tolerance by protecting/stabilizing cellular membranes/enzymes.

Accumulation of phytophenolics, besides proline and soluble sugar, had been reported earlier to maintain cellular homeostasis under abiotic stresses. To examine the effect of salt stress on production of phytophenolics, TPC was measured. Significant increase in TPC in shoot and root, except in case of HD-2329, was in agreement with the findings of Elhamid et al. (2014) in wheat. With constitutively high antioxidant activity and increased TPC, Kharchia-65 could maintain higher antioxidant potential protecting itself from the free-radicals generated under the stress. No significant increase in antioxidant activity/TPC in HD-2329 under salt stress (**Figures 5A–D**) might have resulted into its sensitivity to the stress. Athar et al. (2008) reported production of a wide range of free-radicals in wheat under salt stress, and suggested that higher antioxidant potential contribute in protecting the plant from stress. Strong correlation between antioxidant activity and accumulation of TPC was reported by Rao et al. (2013) and Samota et al. (2017) in rice. Our observations clearly indicate that other components (e.g., ascorbic acid, glutathione, etc.) contributed in the observed antioxidant activity, and TPC also played other protective roles (**Figures 5A–D**).

Shoot and root of the wheat genotypes showed differential responses with respect to the accumulation/distribution of monovalent and divalent cations. As roots are in the direct contact of soil and absorb nutrients, 6- to 16-fold higher accumulation of Na^+^ was observed in root compared to that in the shoot under control. However, under the stress it got transported to or effluxed out of the leaf in case of Kharchia-65 and KRL-210 to maintain its optimum level. Salt-tolerant genotypes possess exclusion mechanisms (regulated expression of HKTs) that control entry of Na^+^ into root (Munns et al., 2006), and efflux of the excessive Na^+^ from photosynthetic tissues (Møller et al., 2009). Our results clearly indicate restricted entry of Na^+^ into roots of Kharchia-65 and KRL-210 (**Figure 6B**), followed by transport of the ion to shoots and its efflux through leaves to maintain bearable level of Na^+^ (**Figure 6A**). HD-2329 and WH-542 probably failed to restrict the entry of Na^+^ into root and efflux of the excessive Na^+^ from leaf.

The adverse effect of salt stress on K^+^ uptake could be seen in the wheat genotypes with the maximum (26%) reduction in absorption/transport was observed in case of HD-2329 (**Figures 6C,D**). Our findings suggest that salt-tolerant genotypes retain selectivity for K^+^ over Na^+^ and maintains lower Na^+^/K^+^ ratio under the stress, while the salt-sensitive genotypes failed to do so (**Figures 6E,F**). It has been suggested that Ca^2+^ is transported from root to shoot during salt stress, and it has been reported that lower Na^+^/K^+^ and Na^+^/Ca^2+^ ratios are associated with salt tolerance in rice (Wu and Wang, 2012). We observed increased Na^+^/K^+^ and Na^+^/Ca^2+^ ratios, particularly in the root tissues of the wheat genotypes (**Figures 6E,F** and Supplementary Table S3). Kharchia-65 could maintain mineral ion homeostasis and confronted least damage due to the stress, while HD-2329 encountered the maximum (**Figure 1** and Supplementary Tables S3). Salt stress is known to affect Mg^2+^ concentration in plant tissues, and lower level of Mg^2+^ in leaf has been correlated with the lower level of chlorophyll in the salt-affected plants (Zhang et al., 2014). We observed a minor but non-significant reduction in Mg^2+^ concentration in shoot and root of the wheat genotypes under the stress (Supplementary Table S3) which is in agreement with the observations of Zhang et al. (2014) who reported minor/non-significant decrease in Mg^2+^ concentration in roots of salt-treated cotton plants even at highest salt (240 mM NaCl) concentration.

Comparative evaluation of salt tolerance ability of the bread wheat genotypes based on a multivariate, comprehensive analysis clearly indicated Kharchia-65 to be more salt-tolerant than KRL-210. Kharchia-65 showed the highest level of chlorophyll, proline, soluble sugar, TPC, antioxidant activity, and maintained lowest level of lipid peroxidation and Na^+^/K^+^ ratio. On the other hand, HD-2329 was identified to be the most salt-sensitive based on the above parameters.

Salt stress causes biochemical, physiological, epigenetic, and several other molecular changes in plants which can be correlated with differential expression of the stress-associated gene(s). Therefore, expression analysis of three *HKT* genes (involved in transport of Na^+^ and/or K^+^) was performed. *TaHKT1;4*, reported to be root-specific (Kumar et al., 2017), was downregulated in Kharchia-65 and KRL-210 under the stress thereby restricted the entry of Na^+^ in root cells (**Figure 7A**). By contrast, the gene was upregulated in HD-2329 and WH-542 which resulted into accumulation of Na^+^ in root (**Figure 6B**) as well as shoot (**Figure 6A**). This might be one of the reasons for the observed salt-sensitive nature of HD-2329 and WH-542. Davenport et al. (2007) reported AtHKT1-directed retrieval of Na^+^ from xylem and its loading into root vacuoles. At cellular level, Na^+^ homeostasis is regulated by controlling Na^+^ entry into root cells, transporting Na^+^ out of shoot cells, and compartmentalizing Na^+^ into vacuoles (Shi et al., 2003).

*TaHKT2;1* and *TaHKT2;3* showed differential expression in shoot and root of the wheat genotypes. These transporters (involved in Na^+^ homeostasis by extruding Na^+^ from root epidermal cells at root-soil interface) were more downregulated in root of Kharchia-65 and KRL-210 under the stress compared to that in WH-542 and HD-2329 genotypes (**Figures 7C,E**). Thus, 2.5-fold downregulated expression of *HKT2;3* in root (**Figure 7E**) can be correlated with minimal increase in Na^+^ accumulation in root of Kharchia-65 (**Figure 6B**). Na^+^ exclusion can also be achieved by net unloading of xylem by parenchyma cells in the stele (Munns et al., 2006) which could be correlated with least accumulation of Na^+^ in shoot of Kharchia-65 under the stress (**Figure 6A**). A similar observation on differential expression of *TaSOS1*, associated with Na^+^ flux from roots to shoots, was reported in durum wheat (Brini et al., 2009).

We were particularly interested to know the role of genetic and epigenetic components in differential expression of the *HKT*s in the contrasting wheat genotypes under salt stress. Root-specific differential expression of *HKT1;4* in the contrasting genotypes (**Figure 7A**) and structural variations (**Figures 10A,C**) could be correlated with salt tolerance ability of Kharchia-65 and hypersensitivity of HD-2329. The point mutations (**Figure 8**), resulting into the loss of ‘Gly-Arg motif’ and ‘selectivity filter’ (**Figure 9**), might be responsible for the observed structural/functional variations in TaHKT1;4.3 (**Figure 10C**). Platten et al. (2006) reported structural analysis of plant HKTs, wherein they observed a conserved “selectivity filter” motif of Ser-Gly-Gly-Gly in case of HKT1 and Gly-Gly-Gly-Gly in case of HKT2. Ion channel are generally bound to the membrane by a highly conserved Gly-Arg motif, which has been reported to be a unique feature of the TrkH transporters of almost all bacterial superfamily of K^+^ transporters (Cao et al., 2011). Considering the importance of these motifs, and their absence in TaHKT1;4.3 (HD-2329), it can be postulated that TaHKT1;4.3 could not perform its functions efficiently. By contrast, HKT2;1 and HKT2;3 showed only minor structural variation (**Figure 11**), but significant difference in their expression in root and shoot of the contrasting wheat genotypes under salt stress. Therefore, the possible role of epigenetic changes in differential expression of the *HKTs* was investigated.

The coding region of *TaHKT1;4* showed only a minor variation in 5mC content with respect to the salt stress, tissues and genotypes (**Figure 12**). More importantly, the variation in 5mC content could neither be correlated with the differential expression of *TaHKT1;4* nor with the salt tolerance level of the wheat genotypes. Context-specific variations in 5mC content in *TaHKT2;1* (**Figure 13**) and *TaHKT2;3* (**Figure 14**) resulted into the appearance of DMR (Supplementary Figure S1). Increase in methylation due to salt stress could be correlated with downregulated expression of the gene. Thus, salt-induced hypermethylation of *HKT2* genes resulted into their downregulated expression. Cytosine methylation in the coding region has been reported to inhibit gene expression (Hohn et al., 1996). Baek et al. (2011) reported that more methylation in CG context in leaf (compared to that in the root), resulted into reduced expression of *AtHKT1* in leaf. However, correlation between cytosine methylation in different contexts and gene expression level has not yet been understood. DNA methylation and histone modifications are influenced by various abiotic and biotic factors, resulting into better adaptability of the plants to the adverse environmental conditions (Kumar, 2017).

This study emphasizes that being a complex trait, salt tolerance is controlled through various (biochemical, physiological, genetic, and epigenetic) mechanisms, and different genotypes have adopted different ways to respond to the stress. Our study demonstrates that Kharchia-65 is one of the most salt-tolerant genotypes acquired genetic/epigenetic mechanisms for controlling *HKT* genes expression, uses physiological and biochemical traits to withstand the stress. The stress is sensed through cell membrane, transduced to various inducers to regulate structural and molecular alterations including H_2_O_2_ accumulation, induction of transcription factors, genetic and epigenetic regulation of gene expression through transcriptional and/or translational reprogramming for protective defense mechanism. Alleles/epialleles for the differentially expressed genes can be identified from the salt-tolerant genotype and validated in EpiRILs/mapping populations for their possible use in the stress breeding program.

## Conclusion

Increasing evidences suggest the key role of genetic background and epigenetic changes in regulating expression of the stress-associated genes (Kumar et al., 2017). Our comprehensive analysis based on agronomical, biochemical, and physiological traits resulted into identification of the most contrasting salt-responsive wheat genotypes. Better membrane stability, antioxidant potential, chlorophyll content, higher accumulation of osmolytes and higher K^+^/Na^+^ ratio under the stress suggested Kharchia-65 to be the most salt-tolerant. By contrast, HD-2329 was found to be highly sensitive to the stress. Expression level of *HKTs* regulated through genetic and epigenetic mechanisms rationalized the observed responses of wheat genotypes. Better understanding about the structural, functional, and regulatory control of HKTs may enable further improvement in salt tolerance of plants in future, and development of more salt-tolerant crop varieties.

## Author Contributions

SK conceptualized, designed and carried out the epigenetic/ionomic/bioinformatic studies, and wrote/revised the manuscript; AB and MA carried out the biochemical and molecular/epigenetic studies, data collection, and their analysis; AS helped in execution of the experimental plan, data analysis, and writing the manuscript.

## Conflict of Interest Statement

The authors declare that the research was conducted in the absence of any commercial or financial relationships that could be construed as a potential conflict of interest.

## Footnotes

1 http://swissmodel.expasy.org/interactive

2 http://swissmodel.expasy.org/

3 www.ncbi.nlm.nih.gov/Structure/cdd/wrpsb.cgi
