# Supplementary material for: Physiological, Biochemical, Epigenetic and Molecular Analyses of Wheat (Triticum aestivum) Genotypes with Contrasting Salt Tolerance
Source: Front Plant Sci. 2017 Jun 30;8:1151. doi: 10.3389/fpls.2017.01151 (PMC5491648; doi:10.3389/fpls.2017.01151)
Supplement: Supplementary file 1 [file Data_Sheet_1.pdf]

Supplementary Table S1. Malondialdehyde level (lipid peroxidation) and total chlorophyll content in shoot under control and salt stressed condition (0, 50, 100, 150, 200, 250 and 300 mM NaCl) in contrasting wheat genotypes at 0, 3, 6, 9, 12, 14 and 16 days after treatment (DAT).

Genotype / DAT	Salinity levels (mM NaCl)													
	0	50	100	150	200	250	300	0	50	100	150	200	250	300
	MDA level (nM)							Chlorophyll content (mg/g FW)						
KRL-210														
3	0.047	0.047	0.048	0.049	0.051	0.053	0.056	2.77	2.65	2.57	2.50	2.48	2.45	2.41
6	0.046	0.047	0.049	0.051	0.053	0.055	0.057	2.75	2.63	2.55	2.48	2.46	2.41	2.35
9	0.046	0.047	0.050	0.053	0.055	0.057	0.060	2.76	2.59	2.53	2.45	2.43	2.35	2.30
12	0.047	0.048	0.051	0.054	0.057	0.060	0.065	2.76	2.57	2.51	2.42	2.40	2.30	2.25
14	0.047	0.049	0.053	0.056	0.060	0.065	0.071	2.75	2.55	2.48	2.38	2.33	2.25	2.17
16	0.046	0.050	0.055	0.061	0.067	0.072	NA	2.75	2.51	2.45	2.32	2.25	2.18	NA
HD-2329														
3	0.047	0.048	0.049	0.050	0.052	0.055	0.059	0.335	0.334	0.321	0.294	0.288	0.272	0.266
6	0.048	0.048	0.050	0.052	0.056	0.057	0.065	0.334	0.333	0.317	0.287	0.282	0.267	0.256
9	0.048	0.048	0.051	0.056	0.063	0.064	0.076	0.335	0.332	0.302	0.280	0.274	0.259	0.247
12	0.047	0.048	0.052	0.059	0.068	0.075	NA	0.334	0.331	0.289	0.271	0.262	0.248	NA
14	0.047	0.049	0.054	0.062	0.076	NA	NA	0.335	0.327	0.278	0.264	0.247	NA	NA
16	0.048	0.051	0.061	0.075	NA	NA	NA	0.335	0.321	0.261	0.249	NA	NA	NA

The values are mean of three replications. NA = Sample not available, because the plant could not survive under the stress condition.

Supplementary Table S2. Membrane stability index of shoot and root tissues of wheat genotypes under control and salt (200 mM NaCl) stress conditions 14 days after treatment.

Genotype	Membrane stability index		
	Control	Salt stress (200 mM NaCl)	Response (%)
Shoot			
Kharchia-65	87.9[b]	78.3[b]	11[a] ↓
KRL-210	86.9[b]	75.6[b]	13[a] ↓
HD-2329	81.4[a]	66.5[a]	18[b] ↓
WH-542	82.5[a]	68.4[a]	17[b] ↓
Root			
Kharchia-65	68.2[b]	55.3[b]	19[a] ↓
KRL-210	67.5[b]	53.3[b]	21[a] ↓
HD-2329	56.8[a]	42.7[a]	25[b] ↓
WH-542	57.6[a]	44.3[a]	23[b] ↓

↓ Indicates reduction in membrane stability index due to the salt stress. Means followed by different *lowercase letters* in a *column* (separately for shoot and root) are significantly different ($P \leq 0.05$) by Fisher's LSD test.

Supplementary Table S3. Effect of salt stress (200 mM NaCl for 14 d) on mineral composition (mg/g DW) in shoot and root of the wheat genotypes.

**Shoot**	Na^+		K^+		Ca^{2+}		Mg^{2+}	
Genotype	Control	Treated	Control	Treated	Control	Treated	Control	Treated
KRL-210	3.33±0.39^b	24.66±1.73^a	60.13±2.36^a	53.63±2.17^b	1.86±0.19^b	1.52±0.08^c	0.74±0.03^a	0.71±0.04^a
Kharchia-65	2.13±0.29^a	21.76±1.66^a	62.67±2.67^a	55.13±1.99^b	2.07±0.13^b	1.78±0.09^d	0.83±0.06^b	0.81±0.05^b
HD-2329	3.13±0.33^b	41.87±1.99^b	58.37±1.76^a	41.67±1.67^a	1.43±0.11^a	1.21±0.06^a	0.66±0.05^a	0.62±0.04^a
WH-542	2.13±0.36^a	39.33±1.66^b	59.37±2.31^a	43.67±1.93^a	1.59±0.09^a	1.36±0.05^b	0.72±0.03^a	0.71±0.05^a
**Root**								
KRL-210	10.66±1.09^a	12.33±1.13^b	46.67±1.91^a	40.16±1.13^b	2.13±0.26^a	1.69±0.17^b	0.76±0.09^a	0.71±0.06^a
Kharchia-65	8.67±0.93^a	9.63±0.97^a	49.26±1.79^a	41.67±1.17^b	2.43±0.29^b	1.75±0.16^b	0.81±0.06^b	0.79±0.04^b
HD-2329	10.98±1.19^a	35.33±1.33^d	46.46±1.89^a	32.32±1.49^a	1.76±0.18^a	1.14±0.11^a	0.67±0.07^a	0.62±0.03^a
WH-542	9.39±0.96^a	27.63±1.03^c	46.23±1.76^a	33.63±1.25^a	1.93±0.19^a	1.27±0.13^a	0.71±0.05^a	0.69±0.04^a

The values are mean of three replications. Means ±SD followed by different *lowercase letters* in a *column* (separately for shoot and root) are significantly different ($P \leq 0.05$) by Fisher's LSD test.

Supplementary Figure S1. Alignment of bisulfite sequences of *TaHKT2;3* gene along with the reference (unmodified) DNA sequences in shoot (Sh) and root (Rt) of Kharchia-65 (Kh) and HD-2329 (HD) grown under control (C) and salt stress (T) conditions.

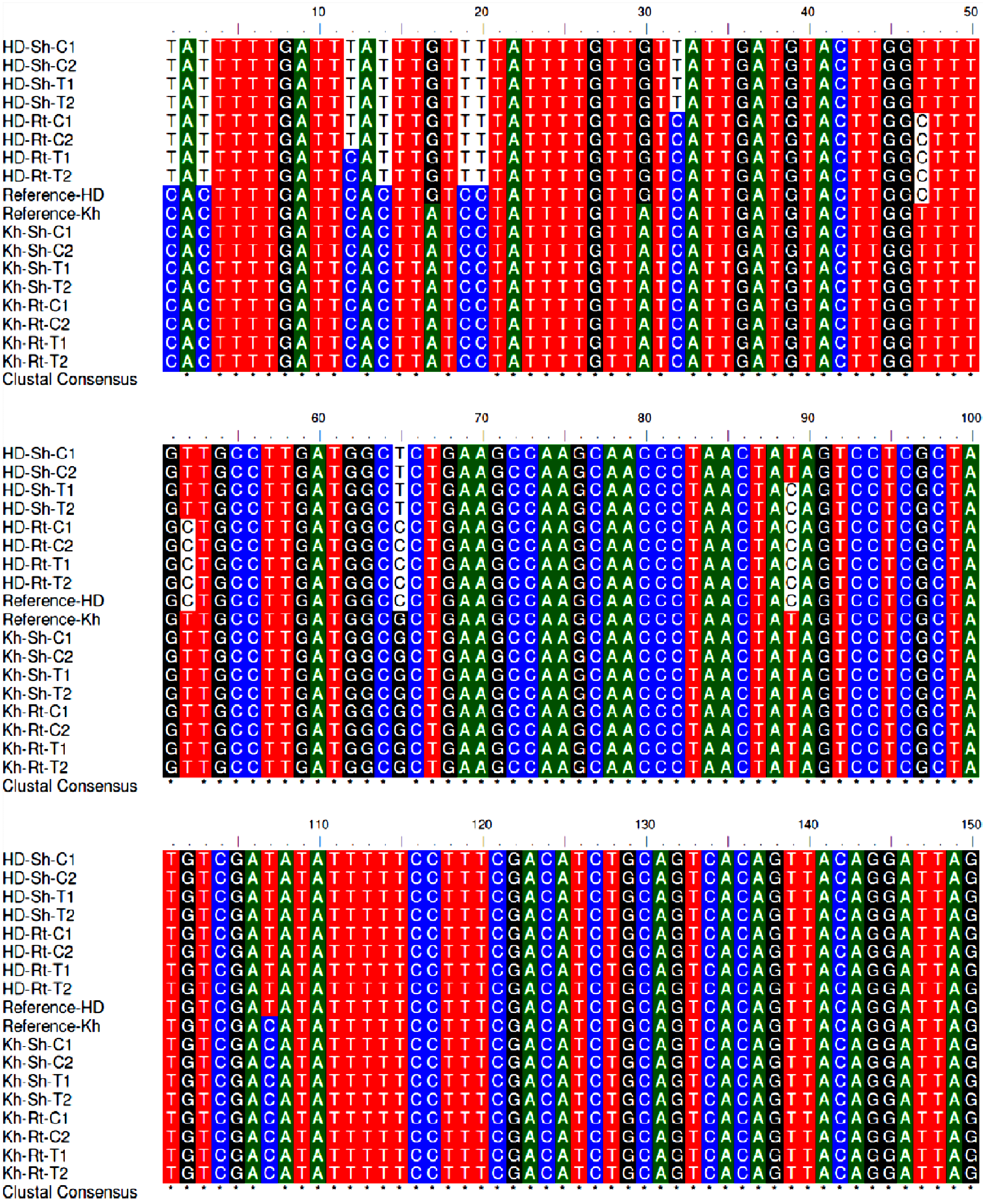

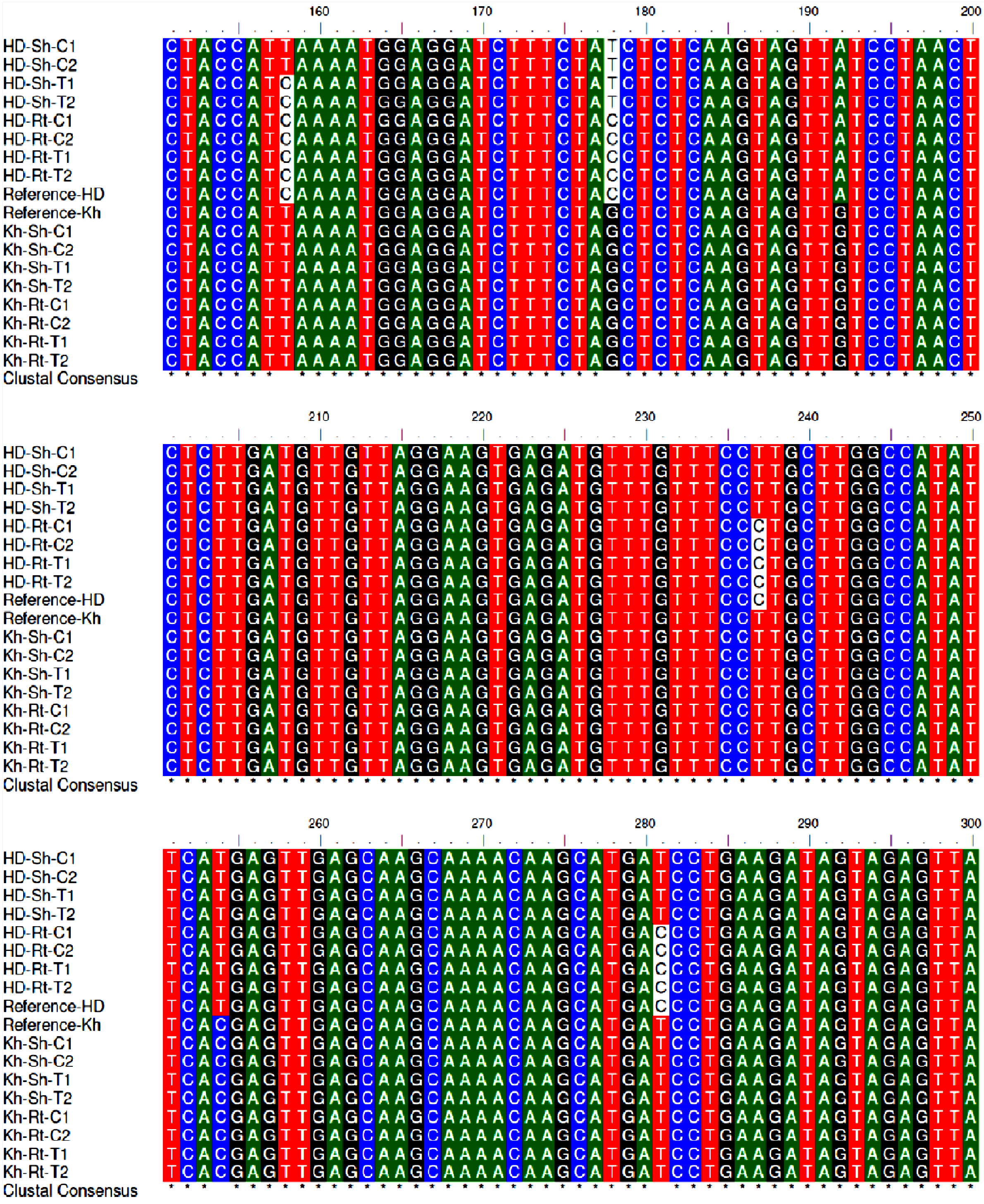

Alignment of only two bisulfate sequencing results for each sample (only 1 – 300 bases) has been presented for the convenience of visualization.
